# The Role of Alcohol in EMS Culture: Perspectives from Frontline Providers

**DOI:** 10.3390/occuphealth1030041

**Published:** 2026-09-10

**Authors:** Maria D. H. Koeppel, Beatrice Shlansky, Nattinee Jitnarin, Christopher K. Haddock, Sara A. Jahnke

**Affiliations:** 1 NDRI-USA, Inc., Leawood, KS 66224, USA; 2 Independent Researcher, Philiadelphia, PA 19104, USA

**Keywords:** alcohol, workplace culture, emergency medical services

## Abstract

Alcohol use among emergency medical service (EMS) providers is an emerging concern given the demanding and stressful nature of the profession, although little research has explored how alcohol fits within EMS culture. This study examined EMS providers’ perceptions of alcohol use and how it intersects with their occupation. Semi-structured interviews were conducted with 31 providers from non-fire-based EMS agencies across the United States. Participants represented a range of licensure levels, agency sizes, geographic regions, and drinking patterns. Interviews explored perceptions of alcohol use, workplace norms, and organizational influences related to drinking. Data were analyzed using an inductive qualitative approach to identify common themes across interviews. Participants described alcohol use as a driving force for social bonding, stress relief, and informal coping with the demands of the job. These findings highlight the importance of considering occupational culture and social norms when developing strategies to address alcohol use and support workforce health in EMS.

## 1. Introduction

Emergency Medical Services (EMS) providers, including paramedics and emergency medical technicians (EMTs), work in high-intensity and unpredictable environments with frequent exposure to trauma, illness, and occupational stress. These demands place EMS personnel at increased risk for adverse mental and physical health conditions, including depression, sleep disorders, obesity, cardiovascular disease, and post-traumatic stress disorder (PTSD) [1–8]. While these outcomes are well documented, alcohol use within the EMS workforce remains comparatively understudied, particularly among providers working in non-fire-based systems.

In the United States, EMS is delivered through two primary models: fire-based and non-fire-based systems. Approximately 30 to 40 percent of EMS is provided through fire departments, where firefighters are cross-trained in EMS, while the majority, 60 to 70 percent, is delivered through non-fire-based systems, including municipal or county agencies, hospital-based services, private companies, and independent providers [9,10]. Although clinical responsibilities are similar, the work environments differ substantially. Fire-based EMS is typically organized around station-based work with stable crews and shared living spaces, whereas non-fire-based EMS is more mobile, with providers often waiting in ambulances between runs and rotating across partners. These structural differences shape social interaction and workplace norms, creating distinct occupational cultures that may influence behaviors such as alcohol use [11,12].

Epidemiological evidence specific to non-fire-based EMS is limited. Much of the existing research includes EMS within broader first responder samples or examines alcohol use indirectly while focusing on other health outcomes [1,13,14]. Alcohol use is often grouped with other substances or analyzed alongside fire-based EMS providers, making it difficult to isolate patterns unique to non-fire-based systems. Available findings suggest that alcohol use is associated with occupational stress and mental health outcomes. For example, excessive alcohol use has been linked to post-traumatic stress symptoms, critical incident stress, and burnout among EMS providers [1,13], and PTSD symptoms have been identified as a predictor of alcohol use in mixed samples [14]. However, reliance on aggregated samples limits conclusions about non-fire-based EMS specifically.

Research on alcohol use in EMS also lags behind the literature on firefighters and law enforcement, where elevated rates of binge drinking and alcohol-related risk are more consistently documented [15–19]. Studies that include EMS suggest similar patterns [15,16], but aggregation with fire-based systems obscures important differences in structural and social environments. Fire-based models emphasize stable crews and shared social space, whereas non-fire-based EMS is less centralized and more variable in team composition. These differences may influence how stress is experienced and how alcohol use is understood, normalized, and enacted, limiting the generalizability of fire-based findings to non-fire-based EMS.

Beyond prevalence and associated outcomes, less is known about the socio-occupational roles alcohol serves within non-fire-based EMS culture. Existing research has largely focused on risk factors [1,13], with limited attention to how providers understand and experience alcohol use in their daily work. Evidence from first responder populations suggests alcohol may function in regulating emotional distress, facilitating social bonding, and processing traumatic experiences [17,19–21]. Whether and how these roles operate within non-fire-based EMS, where social structures differ, remains unclear. These culturally embedded dynamics are not easily captured through quantitative approaches alone.

Given differences in EMS organization and delivery across countries, the present study focuses specifically on the occupational and cultural context of non-fire-based EMS in the United States. Accordingly, this study uses semi-structured qualitative interviews with non-fire-based EMS providers across the United States to examine how alcohol use is perceived, normalized, and integrated into providers’ professional and social lives. By identifying the cultural norms, social dynamics, and contextual factors shaping alcohol use, the findings aim to inform relevant and culturally grounded education and intervention strategies for the non-fire-based EMS workforce.

## 2. Materials and Methods

Data for this analysis were drawn from a National Institutes of Health-funded multi-method study (NIAA#1R01AA030975) examining alcohol use among non-fire-based emergency medical service providers in the United States. The parent study used a sequential mixed methods design to establish foundational knowledge regarding alcohol use in the EMS workforce, including prevalence, risk factors, and consequences. This manuscript reports findings from the qualitative component of the study, which focused on explicating cultural norms, beliefs, and organizational factors that influence alcohol use among EMS providers.

The qualitative interviews were designed to address three aims: (1) to examine the role of alcohol within EMS occupational culture; (2) to identify cultural and organizational factors that may promote or mitigate alcohol misuse; and (3) to explore potential workplace focused policy and practice approaches for encouraging abstention or healthier alcohol use. The study protocol and informed consent procedures were approved by the Institutional Review Board of NDRI USA Inc.

### 2.1. Study Design

We conducted semi-structured key informant interviews with EMS providers using purposive sampling. Recruitment relied on multiple convenience-based strategies to maximize reach and diversity. Recruitment materials were distributed through national EMS organizations, and directly through 13 EMS agencies operating across 15 states. Additionally, recruitment materials were distributed through state EMS offices in six states. State-level participation was based on interest generated through outreach at a national EMS conference attended by representatives from across the United States; the six participating states were those whose representatives expressed interest in actively supporting study recruitment. Recruitment materials included a brief study description and instructions for volunteering to participate. Participants were recruited to reflect variation in geographic region (e.g., west, central, or east) and certification level (e.g., paramedics, emergency medical technicians, and advanced emergency medical technicians). To ensure a range of perspectives related to alcohol use, participants were also screened and categorized into one of three self-reported alcohol use groups: abstainers, moderate drinkers, and heavy drinkers. Based on a self-reported screener using NIH measures [22] abstainers were classified as those who had not consumed alcohol for at least the past 30 days, including those who have never consumed alcohol in their life. Moderate drinkers included those whose average daily intake (based on the number of drinks consumed each week) was not higher than 2 drinks per day for men or not higher than 1 drink per day for women. Heavy drinkers were defined as those who consumed alcohol at rates higher than those classified as moderate. Eligible participants included EMS providers currently working in non-fire-based EMS settings. Recruitment continued until thematic saturation was reached, defined as the point at which no substantively new themes emerged from ongoing analysis.

### 2.2. Interview Methodology

Interviews were conducted between February 2024 and October 2025 by two members of the research team with prior experience conducting qualitative interviews as part of NIH- and FEMA-funded research projects. All interviews were conducted by telephone using a secure conference line to facilitate audio recording. Interviews lasted approximately 30 to 60 min.

Prior to beginning each interview, participants were provided with an overview of the study purpose, confidentiality protections, and voluntary nature of participation, and were given the opportunity to ask questions before providing verbal informed consent. A semi-structured interview guide was used to promote consistency across interviews while allowing flexibility to explore emergent topics. Interview domains included perceptions of alcohol use within EMS culture, workplace norms and expectations, organizational policies and responses related to alcohol, alcohol education and prevention efforts, and informal messaging within EMS organizations.

### 2.3. Participants

A total of 31 interviews were completed and participants included EMS providers across certification levels and organizational roles. Following transcript review and ongoing analytic discussion, the research team determined that thematic saturation had been achieved, as additional interviews were no longer contributing novel concepts or dimensions to the analytic framework.

### 2.4. Data-Analysis Procedures

All interviews were audio-recorded and transcribed verbatim. Transcripts were reviewed for accuracy prior to analysis. Data were analyzed using an inductive qualitative approach informed by grounded theory techniques [23,24]. Analysis focused on identifying recurrent themes, shared and divergent perspectives, and patterns related to cultural norms, organizational context, and alcohol use behaviors.

Researchers first conducted an initial review of complete transcripts to develop a preliminary set of working codes. Two members of the research team independently reviewed all transcripts to familiarize themselves with the data and identify recurring concepts and patterns. Codes were iteratively refined through team discussion and applied to the full dataset. Coding decisions and theme development were driven by the research team using a human-in-the-loop approach, with primary and secondary coders responsible for all code assignment and interpretation.

Following independent transcript review and coding, members of the research team engaged in regular discussions to examine divergent interpretations, challenge emerging assumptions, and refine thematic explanations. Particular attention was given to identifying data that contradicted or complicated developing themes. Because members of the research team brought varying professional backgrounds and levels of familiarity with EMS culture, discussions were used to evaluate whether interpretations were supported by the data rather than individual experiences or expectations. This process helped ensure that themes reflected recurring patterns across interviews while preserving variation in participant perspectives.

To enhance analytic rigor, two primary coders compared coded transcripts and discussed discrepancies. The final analytic framework was used to organize themes and subthemes across interviews. The findings presented here reflect participants’ perceptions and experiences as interpreted through this iterative analytic process rather than objective measures of behavior or prevalence.

To support data organization and retrieval, deidentified transcripts and coded excerpts were managed using qualitative analysis software (Dovetail). Dovetail was used to facilitate data management and generate summary views of coded content; however, all coding, code refinement, and theme development were conducted by the research team. No automated coding was used in the analytic process.

Following completion of coding and theme development, generative artificial intelligence tools (NotebookLM; Google LLC) were used to assist with the retrieval and organization of representative quotations within established coding groups and themes. NotebookLM is a retrieval-augmented generation (RAG) tool that retrieves and synthesizes information from user-provided documents. Deidentified transcripts were uploaded only after coding had been completed and themes had been finalized by the research team. NotebookLM was then queried to identify quotations corresponding to codes and themes that had already been established through human analysis. The software was not used for coding, code refinement, theme generation, interpretation, or analytic decision-making. Because NotebookLM grounds outputs in uploaded source materials and provides citations to the underlying text, it served as a tool for locating and organizing supporting quotations rather than generating findings. To ensure quality control, all quotations identified through NotebookLM were reviewed against the original transcripts and verified for accuracy, context, and consistency with the coded data. Final decisions regarding quotation selection, thematic interpretation, and presentation of findings remained the responsibility of the research team.

## 3. Results

As indicated in Table 1, interview participants included 31 EMS providers from agencies across from western, central, and eastern United States. Respondents were primarily employed in large agencies (200+ providers), with more than half (55%) reporting employment in large organizations, while 22% worked in medium (50–200 providers) and 13% worked in small agencies (<50 providers). Participants reported an average of 15.7 years working in EMS, with experience ranging from 1 to 47 years. Most participants were located in the central region of the country (67%), followed by the western (19%) and eastern regions (13%). Licensure levels varied, with paramedics comprising the largest group (55%), compared to EMT B (25%), EMT A (3%), and other licensure designations (16%). As defined by the National Institute on Alcohol Abuse and Alcoholism (NIAAA), 60% of the group were moderate drinkers, 22% were heavy drinkers, and 16% abstained from alcohol use [22]. The sample was predominantly male (67%), and most participants identified as non-Hispanic (93%) and White (97%). Participant ages ranged from 20 to 72 years, with a mean age of 41.6 years. Overall, the sample was broadly consistent with the national EMS workforce, which is predominantly male and non-Hispanic White, although participants in the present study were somewhat older and less racially and ethnically diverse than national estimates [25].

### 3.1. Occupational Alcohol Culture Themes

Table 2 presents the common themes and subthemes that emerged from the data based on the inductive methods and research objectives. These findings focus on the social, psychological, and evolutionary factors that define the culture of alcohol use among non-fire-based EMS providers. The four main themes include usage patterns and definitions, peer dynamics and social bonding, psychological coping and stress, and cultural shifts and changes over time. Each main theme includes a number of subthemes. The subthemes within each domain provide additional detail on how these broader patterns are experienced and interpreted by EMS providers.

### 3.2. Theme 1: Usage Patterns and Definitions

Overall, the general sense among EMS providers is that alcohol consumption in the profession is likely higher than that of the general public, with the majority of respondents describing a culture where drinking is “rampant” or “above par” due to the intense stressors of the job. While a small subset of providers feel that drinking habits among EMS providers mirror those of the general public, others believe consumption levels are still lower than other male-dominated fields such as construction or firefighting. Additionally, within this cultural landscape, some providers choose to abstain from alcohol entirely, often citing a family history of overuse, previous personal struggles with alcohol, or specific medical conditions as primary drivers for sobriety.

Disagreement about whether alcohol use among EMS personnel is elevated or typical may be partly attributable to respondents’ reliance on subjective definitions of “heavy” or “binge” drinking rather than standardized epidemiologic criteria. Definitions of excessive or binge drinking provided by EMS personnel frequently diverged from clinical or public health definitions. Rather than defining excessive use by the number of drinks consumed within a specified period, respondents often relied on personal physical or cognitive benchmarks, such as blacking out, loss of control, or perceived impairment, to determine whether alcohol use was excessive. When asked about excessive drinking, one moderate drinker shared:

I usually put it as a subjective measurement of if you get to the point of where, “Hey, I was drinking so much I didn’t remember what happened last night” that’s when I think it’s a bit excessive. When you’re drinking to the point of losing control, I guess would be the best way I’d put it ‘cause everyone has different tolerances on what they think is acceptable versus binge drinking. I draw the line at when you lose control of have some gaps in memory from you drinking too much.

Subjective definitions of binge drinking include not drinking “frequently, but when you do, you drink to get hammered”, or “when I hear terms like ‘wrecked’”. While some individuals understand binge drinking to be defined as four to five drinks in one sitting, the overall inconsistency of definitions creates a culture where heavy alcohol use is redefined to mean incapacitation, leading to high-volume but functional drinking (informally defined as drinking that does not interfere with job responsibilities) to appear acceptable and normal within the EMS culture.

In conjunction with idiosyncratic definitions for excessive alcohol consumption, EMS providers often rationalize their alcohol use by comparing their own habits to excessively intoxicated patients. Primarily driven by a “not as bad as” mentality, the daily encounters with alcoholic patients as examples of extreme alcoholism provide a framework against which providers judge their own alcohol use because “we see them, we know them. But we say, ‘It’s not me.’ Which is the typical thing that everyone does… ‘No, no, no. I’m not as bad as XYZ patient.’” This comparison allows providers to normalize their drinking habits as acceptable because “whatever level it is that we’re doing, it’s not that bad because we’ve seen what worse is.” Their justification is reinforced by their subjective definitions that set the bar high for excessive alcohol consumption “because I know what responsible is. I’ve seen worse.”

This double standard around excessive drinking is enhanced by a sense of exceptionalism that EMS providers describe as a forcefield of invincibility. The medical knowledge and professional status create a mentality that “I’m somehow special because…I’m protected. I’ve got a forcefield around me” because providers are trained to know what responsible alcohol use looks like. This perceived protection leads to a belief that negative outcomes related to alcohol use happen to other people, but will not happen to them. This is further amplified by the notion that “we’re supposed to be the public heroes” resulting in “a protective bubble around us of all the social ills in the world.”

These subthemes highlight that alcohol use within EMS is not defined solely by how much or how often providers drink, but also by how they justify those behaviors. Perceptions of excessive drinking are shaped by subjective definitions that elevate the threshold of what is considered excessive, allowing high-volume drinking to be viewed as acceptable when it is perceived as compatible with functioning. This is further reinforced by cognitive dissonance rooted in comparisons to extreme patient cases that normalize their own use. In addition, some respondents described a sense of professional invulnerability, whereby medical knowledge, clinical experience, and occupational identity contribute to the perception that alcohol-related consequences are unlikely to apply to them. Together, these patterns demonstrate how usage patterns and definitions operate within a broader cultural framework that normalizes alcohol use and obscures recognition of risk within the EMS field.

### 3.3. Theme 2: Peer Dynamics and Social Bonding

Throughout the interviews, alcohol is noted as a primary social mechanism and foundational tool for building relationships and team unity within EMS. Alcohol is frequently described as a central element of off-duty gatherings as “anytime that there is a gathering of shift or crew outside of work, there is always drinking involved.” These rituals, which include morning beers after shift change, brunch, celebrations, or general gatherings, serve as time for crews to unwind and solidify their bonds. Strong bonds among crews are essential given EMS shifts may last up to 24 h in a row. These long shifts, in conjunction with alcohol consumption as a “social mechanism and social interaction”, contribute to crew members “becoming your best friends because you end up spending one third to half of your life with these people, so it ends up becoming like a second family.” Close-knit ties and time outside of work, especially after a rough shift, allow for “debriefing about the evening”. In a seemingly bi-directional relationship, alcohol “lowers your inhibitions, so it allows people to bond more easily,” enabling providers to share emotional vulnerability in a way that “all of a sudden becomes a little bit more acceptable because we were at the bar… that’s where I think a lot of processing, talking about calls, talking about trauma and things like that happens. In some ways, that is a safe place to do it.”

Despite alcohol being a key component to developing strong bonds and team unity, its role in social assimilation is much more nuanced, given differences across generations and career stages. Regarding expectations to consume alcohol, one provider did not “feel any direct pressure… no one’s pushing you to do it,”, while another felt “it’s kind of like the good ole boys club that if you don’t do it you’re kind of an outsider.” Choosing not to drink while socializing with crews does not mean “you’re gonna get belittled over it or anything.” While direct pressure may not always be obvious, indirect pressure is “already a part of the culture, so you start to pick up on that. If you hang out with the people that do that, then you’re a part of that group, too, then.” Additionally, “if you’re younger in this line of work, you probably have alcohol as a means of accepting—of assimilation into the culture… would be more peer pressured in that environment.” However, the younger providers who chose not to drink are described as “much as part of our family in EMS as anybody else, whether the alcohol is involved or not involved.” The fear of missing out on social experiences is also noted, regardless of drinking status. Skipping gatherings means feeling “like you missed out on a bunch of stuff ‘cause they had a great time,” highlighting that attendance may carry more weight than drinking itself.

Finally, the data reveal several distinct cultural differences between alcohol use among non-fire-based EMS and fire-based EMS. To begin with, the fire service is described as “almost like a fraternity that you’re getting into…Versus private EMS is more like McDonalds. You go in. You do your shift.” In contrast, “the fire guys tend to get together more than EMS people on our days off. They might crack a beer or two.” As a result, drinking is perceived to be “less amongst EMS than amongst fire. You don’t frequently hear people in the EMS industry talking about how much they drank over the weekend while they were watching sports or going out with friends.” Differences in team structure may contribute to these patterns, as fire stations operate with larger crews (6 to 9 people), while EMS typically operates in pairs of two, where “nobody wants to partner with someone who’s impaired because we depend on each other,” making the effects of heavy drinking harder to conceal.

This dynamic is also reflected in organizational responses to off-duty behavior, as one participant noted that for private EMS companies, “anything that makes ‘em look bad is a threat to losing their contract… In government-owned [fire departments], because it’s not about profit… they’re much more people-centered and so I think there tends to be a higher threshold of forgiveness in those settings.” A small subset of non-fire-based EMS includes flight medics, whose alcohol culture is described as more restrictive, with participants describing themselves as “better-behaved” due to severe consequences. If “we were to mess up that, we’re messing up our whole career…and we face jail time and for putting other people at risk for doing that.” As a result, even during socialization, alcohol plays “a smaller [role]…for us just because of our stringent rules with it.”

Taken together, these subthemes highlight how alcohol functions as a central social mechanism within EMS, facilitating relationship building, bonding, and opportunities for emotional processing outside of work. While alcohol-based gatherings strengthen team cohesion and create space for connection, participation is shaped by complex peer dynamics, with varying degrees of expectation tied to belonging and assimilation. The role of alcohol in social bonding is further influenced by organizational context, including differences between fire-based and non-fire-based EMS. Overall, alcohol use is embedded within a broader social structure in which bonding, identity, and organizational norms influence how and when providers consume alcohol.

### 3.4. Theme 3: Psychological Coping and Stress

When participants are asked to identify why EMS providers may engage in alcohol use, particularly at excessive levels, trauma mitigation and coping are identified as primary drivers. Alcohol is described as a tool to manage the emotional and psychological toll of the job, given that “we see stuff that humans are not meant to see.” It is often used to numb traumatic memories, as providers would “go have a drink to, you know, erase a memory…or something.”, especially after “harder shifts that just can be triggering and wanting to numb yourself away from those bigger calls.”

While alcohol is used to process “big” calls such as pediatric deaths, horrific accidents, or dismemberments, it is also used to manage “repetitive almost microtraumas…It’s the repetitive waking up at 2:00 for XYZ regular frequent flyer again. You know every single shift and sometimes multiple shifts.” or “it’s somebody that’s calling ambulances that doesn’t actually need ambulances and it’s not traumatic, but it’s just this chronic stressor…it does build after a while.” In general, when EMS providers lack other healthy mechanisms to manage stress, “they’ll try to use drugs or alcohol to help with that decompression as a last resort. Then it quickly becomes a first resort ‘cause there’s no other way.” This pattern becomes ingrained, and providers may not “know 100 percent that you’re doing it in order to cope, I think that we do that subconsciously.”

Related to trauma mitigation, “hydraulic debriefing” is described as an informal process in which providers gather after shifts to verbally process traumatic calls, with alcohol acting as an emotional lubricant. These settings are described as opportunities to “blow off that steam. Talk about those stressful moments…you might do some hydraulic debriefing.” These environments, often involving coworkers and alcohol, are perceived as safe spaces because “being in a bar and people who are drinking, that provided a little bit of cover, if you will, for those people to have those big emotions.” Given its ability to lower inhibitions, alcohol “makes it easier to be vulnerable, to talk about stuff with your coworkers, ‘cause usually maybe you wouldn’t be as open as after having a couple beers.” This may be especially true for men, as “sometimes it’s easier for men maybe to be in that vulnerable place with a beer”. These accounts suggest that “hydraulic debriefing” functions as an informal, culturally accepted space for processing trauma, where vulnerability is facilitated by the presence of alcohol.

Finally, alcohol use among EMS providers is described as a functional tool for physiological and psychological regulation in response to the demands of the profession, beyond that of singular traumatic events. As previously noted, the job involves “a level of tension…alcohol is an easy way to reduce that, and especially since it’s socially acceptable,” providing a way “to help numb a memory or a feeling or an interaction.” More than a coping mechanism, alcohol is used to help providers get “back into the feeling of just being a person again instead of being a first responder.” In a work environment characterized by uncertainty and limited control, alcohol is perceived as providing a sense of control: “I’m gonna drink to make myself feel better” because “I can’t control the losses,…but you can control picking up a bottle… You can control putting yourself into that position and then know, by the way, you do get the mental relief of not being able to focus on that.”

Additionally, alcohol is described as aiding sleep, as one provider noted, “didn’t sleep at all last night, so I’m gonna have a few drinks so I can help myself sleep.” Overall, alcohol’s functional role is reflected in its perceived ability to reduce physiological arousal, particularly in a workforce that “attracts people who are pretty high-sensation seeking and who are looking to do things, and sometimes I think they need to quiet down their nervous system a little bit… I really think ultimately people do it in EMS ‘cause it quiets down their nervous system.”

These findings highlight that alcohol use among EMS providers is closely tied to the psychological demands of the profession, serving as a primary mechanism for coping with both acute trauma and chronic stress. Whether in response to “big” calls or the accumulation of everyday stressors, alcohol is used to numb, decompress, and manage emotional strain, sometimes becoming a subconscious coping strategy. Practices such as hydraulic debriefing reinforce alcohol’s role as a socially accepted context for processing difficult experiences and expressing vulnerability. Beyond emotional coping, alcohol is also used as a functional tool to regulate mood, facilitate sleep, and restore a sense of control in an otherwise unpredictable work environment.

### 3.5. Theme 4: Cultural Shifts and Evolution

Data from participants highlight that the EMS profession is currently experiencing a cultural transformation, moving away from a historical “work hard, play hard” ethos toward a more health-conscious professional identity. A prominent driver of this shift is the integration of a new generation into the workforce. Veteran providers are associated with a culture in which alcohol is a primary tool for coping with trauma and building camaraderie, as “there was a lot more drinking going on than I see in this current generation of providers…it was more acceptable.” In contrast, younger providers are viewed as more health-conscious, both physically and mentally. While younger EMS providers do engage in alcohol use, “this next generation of providers that I’ve seen out there, they’re more responsible,” potentially reflecting “a culture of just sort of physical fitness and healthy eating.” This generational shift is also reflected in social activities outside of work. While “people who are my age or older, …their number one is, ‘Let’s go have a couple drinks, then we can talk.’… the younger is, ‘You want to go to coffee? You want to go get lunch?’ That’s the difference.” Among younger providers, alcohol no longer appears to be the primary coping mechanism, as “we look at the younger group…I don’t feel that’s what they’re going to first…I think that they have a little bit more ability to talk things out to cope.”

This shift in health-related values reflects a broader change in attitudes toward well-being across the field, as “it seems more people are focused on well-being in general.” Supported by a younger workforce that feels “more comfortable reaching out in regards to mental health resources as opposed to… typically people over 40,” there is increasing awareness and acceptance of mental health support. As the EMS field continues to evolve, it is described as becoming more professional, with “more awareness about alcohol abuse,” resulting in a decreased acceptance of showing up to work hung over, whereas previously, if “you came in a little hung over. No one cared. Now the environment’s changed a little bit.” Although processing work-related trauma without alcohol is becoming more normalized because “it’s a normal thing to want to talk about the shit show that you just got done dealing with,” some providers continue to report stigma associated with seeking help, as “admitting that you have a problem…that makes you weak,” and “it might not be well received because somebody admits to having a problem or even needing to take a mental health day for whatever reason.” In response, some agencies have developed confidential peer support teams that allow providers to seek help from colleagues who understand their experiences, where “you don’t even have to go to your supervisors and stuff.”

A final shift is observed in patterns of substance use, particularly movement away from alcohol as a primary means of decompression and emotional downregulation. Younger providers are described as increasingly using caffeine and high-potency energy drinks to manage job demands. “I always see them [younger EMTs] with an energy drink or a coffee,” leading one participant to note “I’d be more concerned about the abundant use of energy drinks, coffee, vaping, other forms of stimulation.” Additionally, participants expressed concern about increasing use of THC and cannabis products, with one noting, “I worry that there are people who are now just using cannabis rather than alcohol.” This trend may continue “because it’s so accessible, I think we’ll see an incline in maybe marijuana use as well. Just because it’s more accessible now.” Cannabis is described as appealing because “the younger crowd doesn’t think marijuana is wrong. It doesn’t give you [a] hangover.” This shift presents challenges for organizations, as “if you have cannabis in your system…they cannot prove when you took it. They can’t prove that it was impairing your abilities,” whereas “alcohol is one of our more easily detectable substances of abuse.”

Overall, these findings suggest that the culture of alcohol use within EMS is evolving alongside changes in workforce composition, health awareness, and professional expectations. While alcohol has historically played a central role in coping and socialization, a younger generation is contributing to more health-conscious approaches and greater openness to mental health support, despite ongoing stigma. At the same time, this shift does not necessarily reflect reduced substance use, but rather a transition toward alternatives such as caffeine, energy drinks, and cannabis in young EMS personnel. These patterns reflect a profession in transition, in which traditional norms around alcohol are being redefined.

## 4. Discussion

This study examined how alcohol use is perceived, understood, and integrated into the professional and social lives of non-fire-based EMS providers. Findings suggest that participants perceived alcohol to be deeply embedded within the occupational culture of EMS and to serve functions that extend beyond individual drinking behavior. Participants described alcohol as a means of coping with occupational stress, facilitating social connection, and processing difficult and traumatic experiences with coworkers. These findings suggest that participants viewed alcohol use as being shaped not only by repeated exposure to stress and trauma, but also by shared norms and beliefs that make drinking a culturally accepted and organizationally tolerated means of coping and informal debriefing. In this way, participants described alcohol as functioning not only as an individual behavior, but also as a substitute for formal recovery and mental health support structures that may be limited or inconsistently available within EMS organizations. Participants also described signs that these norms may be changing, with younger providers perceived as less centered on alcohol and more open to discussing mental health, while increasingly relying on other substances, such as energy drinks, cannabis, and vaping products, to manage the physical and psychological demands of the job. These observations reflect participant perceptions and experiences and should be interpreted as hypothesis-generating findings that warrant further investigation in larger samples of EMS personnel.

These findings are consistent with a substantial body of research demonstrating elevated rates of alcohol use and coping-motivated drinking among firefighters and law enforcement personnel. Studies of firefighters have documented high rates of binge drinking and problematic alcohol use, with alcohol often described as a means of unwinding after shifts, promoting social cohesion, and managing exposure to traumatic events [17,20,21,26,27]. Similarly, research in law enforcement has shown that both operational stress and organizational stress are associated with more frequent binge drinking, suggesting that alcohol use is shaped not only by critical incident exposure but also by workplace culture and administrative pressures [28,29].

The present study extends this literature by situating alcohol use within the specific context of non-fire-based EMS, a workforce that has often been combined with firefighters or other first responders in prior studies. Participants described many of the same functions identified in firefighter and police research, including coping with trauma, facilitating social bonding, and reducing emotional distress. At the same time, participants described alcohol as an informal means of debriefing and emotional processing, which may be particularly important in non-fire EMS systems with limited access to stable crews, station-based social structure, and formal support mechanisms more common in fire departments. Participants described features of non-fire EMS, including rotating partners, mobile work environments, and less consistent access to shared physical spaces, that may increase reliance on informal and off-duty opportunities for social connection and emotional processing. Qualitative interviewing further identified several cultural processes through which participants perceived alcohol use as becoming normalized, including subjective definitions of risky drinking, comparisons with severely intoxicated patients, and beliefs that medical knowledge confers protection from harm. These mechanisms are difficult to detect in prevalence studies and provide insight into how occupational culture may shape alcohol use within EMS.

A key contribution of this study is the insight qualitative interviewing provided into how participants perceived alcohol use as becoming normalized within EMS culture. Whereas prevalence studies can estimate how often EMS providers drink or meet criteria for hazardous alcohol use, they offer limited insight into how providers interpret and justify these behaviors. Participants in the present study described definitions of problematic drinking that differed substantially from public health guidelines, with alcohol use viewed as concerning primarily when it resulted in blackouts, loss of control, or visible impairment rather than when it exceeded established binge-drinking thresholds. Participants also appeared to minimize perceived risk through downward social comparison, contrasting their own drinking with the severe alcohol misuse encountered among patients. Participants also described a form of professional exceptionalism in which medical training and repeated exposure to substance-related harm fostered a belief that EMS clinicians were better able than the general public to recognize and control problematic use. Finally, alcohol was described as creating a socially acceptable space for discussing difficult calls and processing emotional experiences with coworkers. Participants described drinking as serving not only as a coping behavior, but also as an informal debriefing mechanism within EMS culture. At the same time, participants described evidence that these norms may be changing as younger providers were perceived as more open to discussing mental health and seeking support, and increasingly likely to use alternative substances, including energy drinks and cannabis, to manage the demands of the profession.

Several limitations should be considered when interpreting these findings. First, the study was based on a purposive sample of 31 non-fire-based EMS providers and was designed to capture depth and diversity of perspectives rather than produce nationally representative estimates. Recruitment relied on voluntary participation and convenience-based recruitment strategies, which may have introduced selection bias. Individuals with particularly strong opinions, experiences, or concerns related to alcohol use may have been more likely to participate, while those who were uncomfortable discussing alcohol or perceived the topic as sensitive may have chosen not to participate. As a result, the findings should be viewed as reflecting the perspectives of those who volunteered rather than representing the views of the broader EMS workforce. Second, the sample was predominantly White and male, somewhat older and less racially and ethnically diverse than the national EMS workforce, and disproportionately represented providers from the central United States and larger EMS agencies. Alcohol-related norms, coping practices, and attitudes toward mental health may vary across demographic groups, geographic regions, and organizational settings; therefore, these sample characteristics may limit the transferability of findings to the broader U.S. EMS workforce. Third, all data were self-reported and may be affected by recall bias or social desirability, particularly given the sensitive nature of alcohol use. Additionally, because the study was qualitative and cross-sectional, the findings are intended to generate insight into cultural and organizational processes rather than establish causal relationships. Future research using larger quantitative and mixed-methods samples is needed to determine the prevalence and generalizability of these themes within the broader EMS workforce. Finally, as with all qualitative research, findings were shaped by participant accounts and researcher interpretation, although multiple coders and iterative team discussions were used to strengthen analytic rigor.

The findings of this study suggest several potential implications for occupational health practice within EMS organizations. Because participants described alcohol as serving as both a coping mechanism and an informal means of debriefing, efforts to reduce alcohol-related risk should focus not only on individual behavior, but also on the underlying occupational needs that alcohol is helping to meet. These findings suggest that agencies may benefit from creating structured, alcohol-free opportunities for peer connection and post-incident discussion, allowing providers to process difficult experiences and maintain social support without relying on drinking-centered gatherings. Confidential pathways to mental health and substance use services, including peer support teams and external counseling resources, may also help address barriers to help-seeking, particularly in environments where stigma remains a concern. The findings also support consideration of education for supervisors and field training officers on recognizing signs of problematic substance use, responding to mental health concerns, and modeling healthy coping behaviors. Onboarding and continuing education programs may also provide an opportunity to address the cumulative effects of trauma, fatigue, sleep disruption, and substance use, while setting realistic expectations about the emotional demands of EMS work. The findings also suggest the importance of clear organizational policies regarding impairment, including expectations related to reporting to work under the effects of alcohol, cannabis, or residual hangover symptoms that may compromise performance. As a result, these findings suggest that effective prevention efforts should extend beyond alcohol-specific education and instead strengthen the organizational supports needed for recovery, connection, and psychological well-being. Addressing alcohol use in EMS may therefore benefit from organizational strategies that complement individual-level approaches by providing healthier alternatives for connection, recovery, and psychological support.

## Figures and Tables

**Table 1.** Participant Characteristics.

	N (Mean)	% (Range)
**Sex**		
Female	10	32.3%
Male	21	67.7%
**Ethnicity**		
Hispanic	2	6.5%
Non-Hispanic	29	93.5%
**Race**		
White	30	96.8%
Asian	1	3.2%
**Age (years)**	41.6	20–72
**Size of Agency**		
Large	17	54.8%
Medium	7	22.6%
Small	4	12.9%
Unsure	3	9.7%
**Time in EMS (years)**	15.7	1–47
**Region**		
East	4	12.9%
Central	21	67.7%
West	6	19.4%
**Licensure level**		
EMT B	8	25.8%
EMT A	1	3.2%
Paramedic	17	54.8%
Other	5	16.1%
**Drinking**		
Abstainer	5	16.1%
Moderate	19	61.3%
Heavy	7	22.6%

**Table 2.** Thematic Summary.

Main Themes	Sub-Themes
Usage patterns and definitions	Overall use patterns Subjective definitions Cognitive dissonance Forcefield of invincibility
Peer dynamics and social bonding	Social glue and team camaraderie Social assimilation Uniqueness to non-fire EMS
Psychological coping and stress	Trauma mitigation and coping Hydraulic debriefing “Functional” utility
Cultural shifts and evolution	Generational divide Mental health awareness Substance substitution

## Data Availability

Data are available from the corresponding author upon reasonable request.
